# Research on Omnidirectional Stereo Measurement Using Convex Mirrors and Vertical Disparity

**DOI:** 10.3390/s23063243

**Published:** 2023-03-19

**Authors:** Yuki Ozawa, Shingo Kimura, Yiling Zhu, Atsutoshi Kurihara, Yue Bao

**Affiliations:** Division of Informatics, Graduate School of Integrative Science and Engineering, Tokyo City University, Tokyo 158-8557, Japan; g2181432@tcu.ac.jp (S.K.); g2181499@tcu.ac.jp (Y.Z.); g2291401@tcu.ac.jp (A.K.); bao@g.tcu.ac.jp (Y.B.)

**Keywords:** distance measurement, depth sensing, stereo camera, omnidirectional image, omnidirectional measurement

## Abstract

We propose an omnidirectional measurement method without blind spots by using a convex mirror, which in principle does not cause chromatic aberration, and by using vertical disparity by installing cameras at the top and bottom of the image. In recent years, there has been significant research in the fields of autonomous cars and robots. In these fields, three-dimensional measurements of the surrounding environment have become indispensable. Depth sensing with cameras is one of the most important sensors for recognizing the surrounding environment. Previous studies have attempted to measure a wide range of areas using fisheye and full spherical panoramic cameras. However, these approaches have limitations such as blind spots and the need for multiple cameras to measure all directions. Therefore, this paper describes a stereo camera system that uses a device capable of taking an omnidirectional image with a single shot, enabling omnidirectional measurement with only two cameras. This achievement was challenging to attain with conventional stereo cameras. The results of experiments confirmed an improvement in accuracy of up to 37.4% compared to previous studies. In addition, the system succeeded in generating depth image that can recognize distances in all directions in a single frame, demonstrating the possibility of omnidirectional measurement with two cameras.

## 1. Introduction

### 1.1. Backgrounds

In recent years, there has been a great deal of research and development on driver assistance systems for automobiles, fully automated driving, and unmanned search robots for disaster areas. As representative examples of autonomous driving technology, we will introduce the unmanned vehicle “R2” [1], the serving robot “BellaBot” [2], and the electric vehicle “e-palette” [3].

The “R2” [1] is an unmanned vehicle developed by Nuro, an American self-driving technology company. They launched a service to transport medical equipment, medicines, food, and beverages to assist medical personnel responding to a new type of coronavirus infection (COVID-19).

The BellaBot [2] is a cat-shaped meal delivery robot invented by Pudu. It can provide a stable, non-contact food delivery service and has already been introduced in many restaurants in Japan.

The “e-Palette” [3] is an electric vehicle developed by Toyota Motor Corporation exclusively for Autono-MaaS (autonomous mobility as a service). As a Worldwide Mobility Partner of the Olympic and Paralympic Games, Toyota provided more than a dozen e-Palettes (Tokyo 2020 specifications), the first Toyota electric vehicles dedicated for Autono-MaaS, to support the transportation of athletes and Games officials as buses that traveled around the athlete village. The e-Palettes were used as buses to transport athletes and Games officials around the village.

In these fields, forward distance measurement and three-dimensional measurement of the surrounding environment are indispensable. There are various types of sensors, but depth sensing using cameras is the most widely used and one of the most important sensors for recognizing the surrounding environment. Depth sensing with cameras has been studied for a long time and is widely used because of its high measurement density, wide measurement range, and diversity of information obtained.

### 1.2. Existing Technology

Depth sensing using cameras is often based on a technique called stereo matching, which uses two cameras to calculate the disparity between two corresponding pixel regions from a pair of images to obtain the distance [4]. There have been many studies on distance measurement by stereo matching, especially for automatic guidance. In such cases, a wider range of measurements is important.

In the study of Hirotaka Iida et al. [5], a fisheye lens was used as a stereo camera for distance measurement, to enable a wider range of measurements that are impossible with ordinary cameras. However, the angle of view of a fisheye lens is generally about 180°, which restricts the measurement range.

In the study by Changhee Won et al. [6], the team addressed the limitation of Iida et al. [5] by utilizing four fisheye cameras and measuring the distance in all directions. Although this method allows for stable measurement in all directions, it requires double the number of cameras compared to the method of Iida et al. [5]

Schönbein, Miriam et al. proposed a method [7,8] in which omnidirectional visual sensors are installed in front of the vehicle roof and on the left and right sides. In this method, two cameras are used for omnidirectional measurement, but the accuracy of the measurement is not sufficient because one of the sensors is reflected in the image, and the large difference between the images makes matching from the image development difficult.

Aoki et al. [9] used a stereo omnidirectional camera consisting of two omnidirectional cameras to estimate depth. In estimating depth, they extract feature points from the least distorted portion of the fisheye image captured by the omnidirectional camera. However, their method has the following problems: depth is estimated only from the least distorted part of the image, and it is difficult to estimate the depth of the entire circumference at the same time due to the distortion of the fisheye lens. Moreover, extracting feature points is difficult in their method.

**Figure 1:**
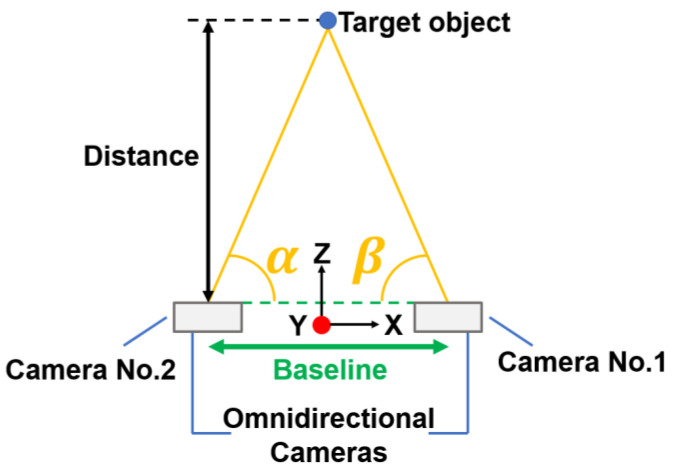
Image of the method of Tanaka Shunya et al.

In the study by Tanaka Shunya et al. [10], a commercially available all-sky camera [11] was utilized to perform object detection through machine learning and azimuth angle calculation to achieve omnidirectional measurement using two cameras. However, this method faced a drawback as it resulted in a blind spot in the camera’s extended baseline (Figure 1) that could not be detected, so it was not a perfect omnidirectional measurement. Despite capturing an omnidirectional image by the back-to-back method, the fisheye camera lens faced the issue of chromatic aberration. To eliminate this chromatic aberration, multiple lenses must be stacked. Since it is impossible to mount multiple lenses on a small all-sky camera, it is impossible to eliminate chromatic aberration. Therefore, we considered that other omnidirectional imaging methods should be used to achieve more accurate measurements.

This research uses a method with no blind spots by using an omnidirectional camera with a convex mirror, which in principle does not cause chromatic aberration. The mirror used is a hyperbolic mirror. This technique was adopted because it is the most superior in terms of visibility in measurement among various omnidirectional imaging techniques and matches the conditions of our method. The measurement using hyperbolic mirrors is a new method using vertical disparity by installing cameras at the top and bottom of the image. This method solves the problem of conventional methods, where a complete omnidirectional measurement is not possible because the other camera is also included in the image. The purpose of this research is to detect obstacles and to create a sensor that can be used for obstacle detection with automatic guidance, similar to the conventional stereo method. To demonstrate the usefulness of this method, we conducted a measurement experiment to verify the measurement accuracy and a depth image generation experiment to verify whether measurement in all directions is possible. In the measurement experiment, the results were compared with those of the conventional method to verify the accuracy. The conventional method compared was Tanaka’s method [10], which was selected as the one with the best measurement accuracy among the conventional studies that have performed measurements under similar conditions. While the conventional method had a relative error of up to 40%, the new method succeeded in reducing the error to a maximum of 2.6%. In addition, when comparing the relative error for each distance, the accuracy was better than that of the conventional method at all points.

## 2. Materials and Methods

### 2.1. Proposed System Configuration

**Figure 2:**
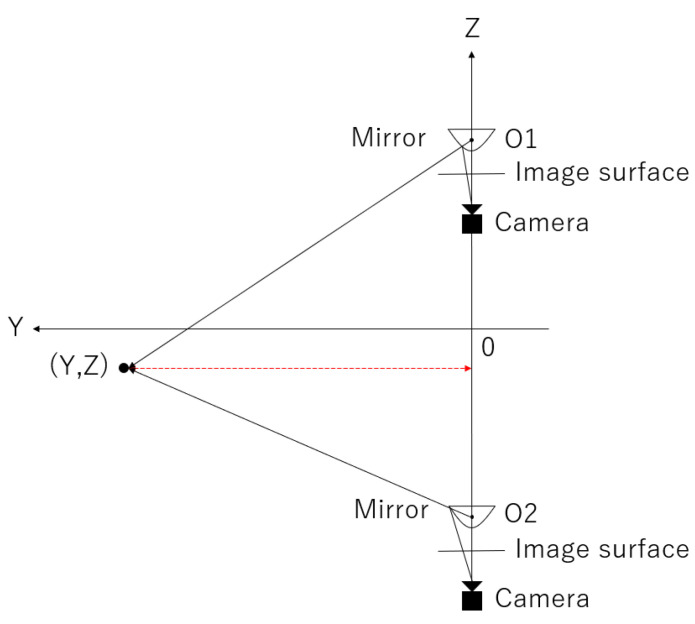
Conceptual diagram of the proposed system.

A conceptual diagram of the proposed method is shown in Figure 2. The proposed system consists of two convex mirrors and two cameras. The camera is fixed vertically upward and a convex mirror is attached to the camera lens. By capturing the image reflected in the convex mirror from directly below, it is possible to capture an omnidirectional image in one shot. This device is henceforth referred to as the omnidirectional visual sensor. Another similar device is installed vertically. By combining the images captured by these two omnidirectional visual sensors, it is possible to achieve distance measurement and depth image generation in all directions.

### 2.2. Omnidirectional Visual Sensor

**Figure 3:**
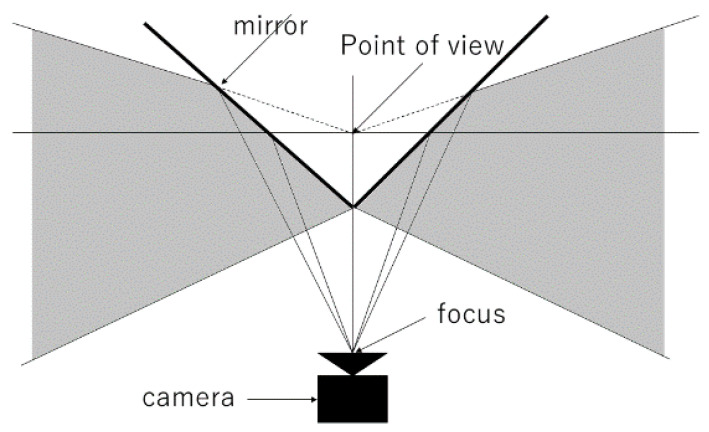
Conical mirror.

The omnidirectional visual sensor is described below. As shown in Figure 2, the omnidirectional visual sensor consists of a convex mirror and a camera facing vertically upward and can capture all directions in a single shot. Three types of possible convex mirrors were considered: conical, spherical, and hyperbolic. As shown in Figure 3, the method using a conical mirror provided high lateral resolution, but reflected light rays from below did not enter the lens, making it difficult to capture the feet.

**Figure 4:**
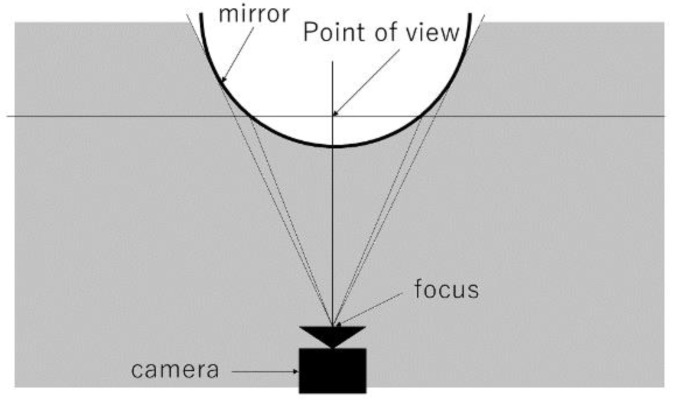
Spherical mirror.

As shown in Figure 4, in the method using a spherical mirror, the closer one gets to the outer edge of the mirror, the larger the area to be imaged concerning the area to be projected, resulting in good resolution of the feet, but the camera itself contains a large area, and objects in the distance or on the side are not well captured as if compressed.

**Figure 5:**
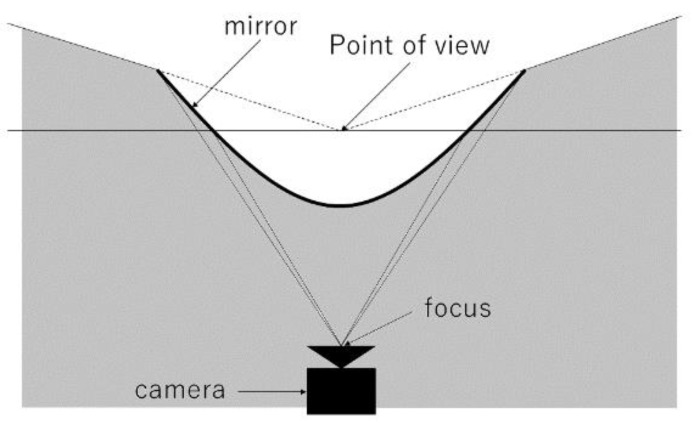
Conceptual diagram of the omnidirectional visual sensor.

In contrast, hyperbolic surfaces offer a distinct advantage, as shown in Figure 5, as the upper field of view has the same high resolution as the method using a conical mirror while avoiding the limitations of the lower field of view found in spherical mirrors. Therefore, the hyperbolic surface has the advantage in the field of view of both methods using conical and spherical mirrors in that it is side-centered and also provides a foot view. Therefore, hyperbolic mirrors are used in the present system.

### 2.3. Panoramic Expansion

**Figure 6:**
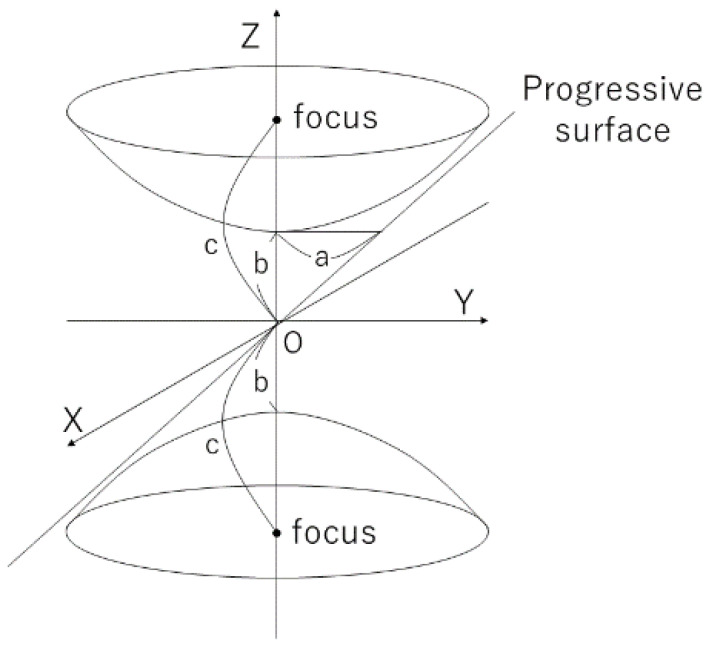
Bilobed hyperbolic surface.

Since a hyperbolic mirror is used, distortion occurs during imaging. In addition, since we want to use vertical epipolar lines for measurement, this method performs panoramic expansion on an omnidirectional image. First, the optics of the hyperbolic mirror is described. A 2-leaf hyperbolic surface is used for the hyperbolic surface in this method. As shown in Figure 6, a 2-leaf hyperbolic surface is a surface obtained by rotating a hyperbola around (Z-axis). The characteristic of the hyperbola, which has two foci $\left(c=\sqrt{a^{2}+b^{2}}\right)$, (0, 0,+c) and (0, 0,−c), is also retained in the hyperbolic surface. Moreover, as shown in Figure 5, consider 3-dimensional coordinates O-XYZ with the Z-axis as the vertical axis. In this case, the 2-leaf hyperbolic surface can be expressed by the following equation.

(1) $$\frac{X^{2}+Y^{2}}{a^{2}}-\frac{Z^{2}}{b^{2}}=-1$$

Note that a and b are constants that define the shape of the hyperbola. In this method, the hyperbolic surface in the region of Z>0 among the two leaves is used as a mirror.

**Figure 7:**
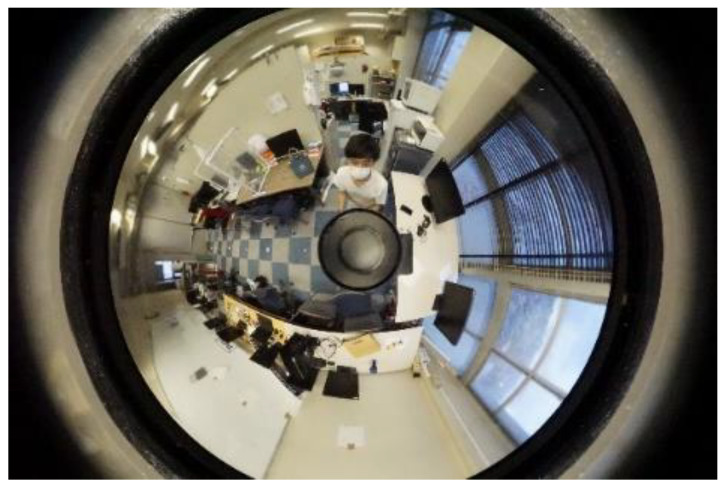
Omnidirectional image.

Next, the method of developing an omnidirectional image used in this method into a panoramic image is explained using an actual photograph as an example. Figure 7 is an omnidirectional image taken.

**Figure 8:**
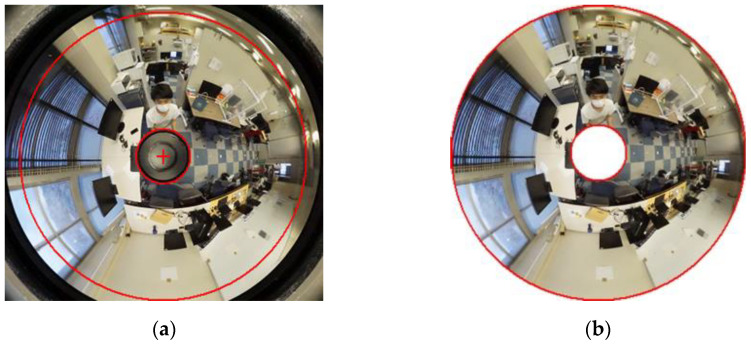
Crop image: (**a**) before cropping; (**b**) after cropping.

First, to extract only the area to be used for measurement, an omnidirectional image taken is cropped in the area surrounded by red as shown in Figure 8a,b. The area to be cropped is determined according to the shooting range of the camera used for the measurement.

**Figure 9:**
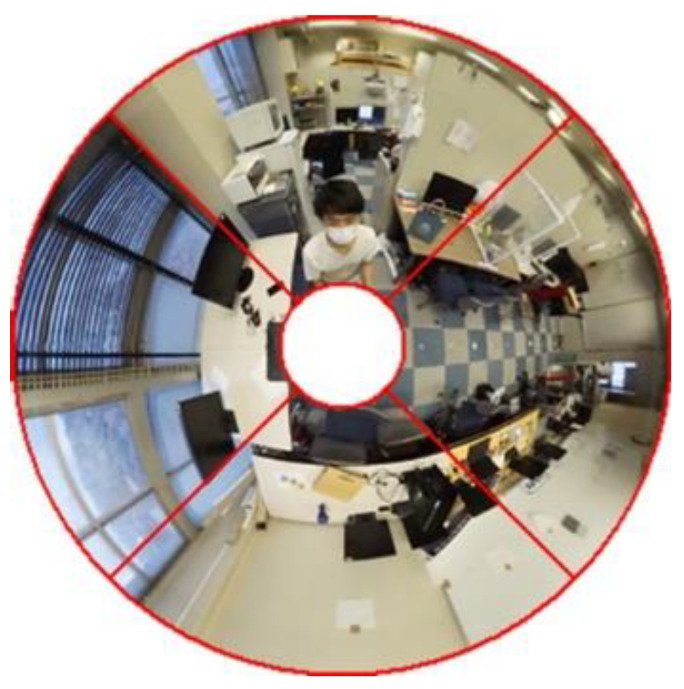
Quadranting the image.

Next, the image is divided into four equal parts as shown in Figure 9.

**Figure 10:**
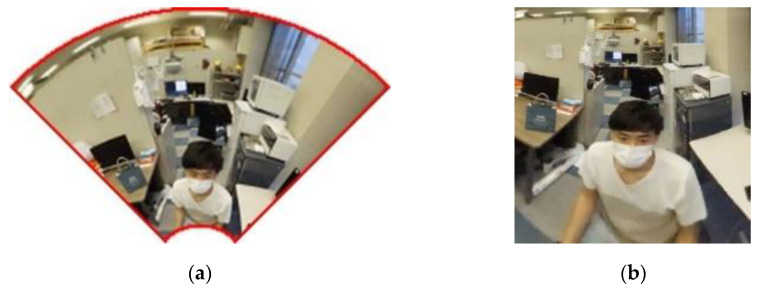
Cropping image: (**a**) image before expansion; (**b**) image after expansion.

Next, a perspective projection transformation is performed on each quadratically divided image using the following equation. The panoramic expansion of an omnidirectional image using a hyperbolic mirror can be expanded using the following equations [12].

(2) $$\begin{array}{c} x=-\frac{a^{2}fX}{\left(b^{2}+c^{2}\right)Z-2bc\sqrt{X^{2}+Y^{2}+Z^{2}}}+x_{c} \end{array}$$

(3) $$\begin{array}{c} y=-\frac{a^{2}fY}{\left(b^{2}+c^{2}\right)Z-2bc\sqrt{X^{2}+Y^{2}+Z^{2}}}+y_{c} \end{array}$$

where X, Y, Z are points in three-dimensional coordinates; *x*, *y* are points in the image coordinate system; $x_{c}$, $y_{c}$ are the image center coordinates; a, b, c are the mirror parameters of the hyperbolic surface; and f is the focal length. Since the mirror parameters and focal length are known, expansion is possible. The results of the expansion of the quadratically divided image are shown in Figure 10a,b.

**Figure 11:**
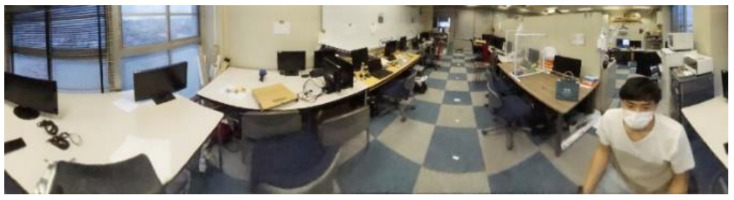
Panoramic image.

Finally, as shown in Figure 11, the panoramic image is completed by connecting the four developed images.

### 2.4. Vertical Disparity Stereo Matching

**Figure 12:**
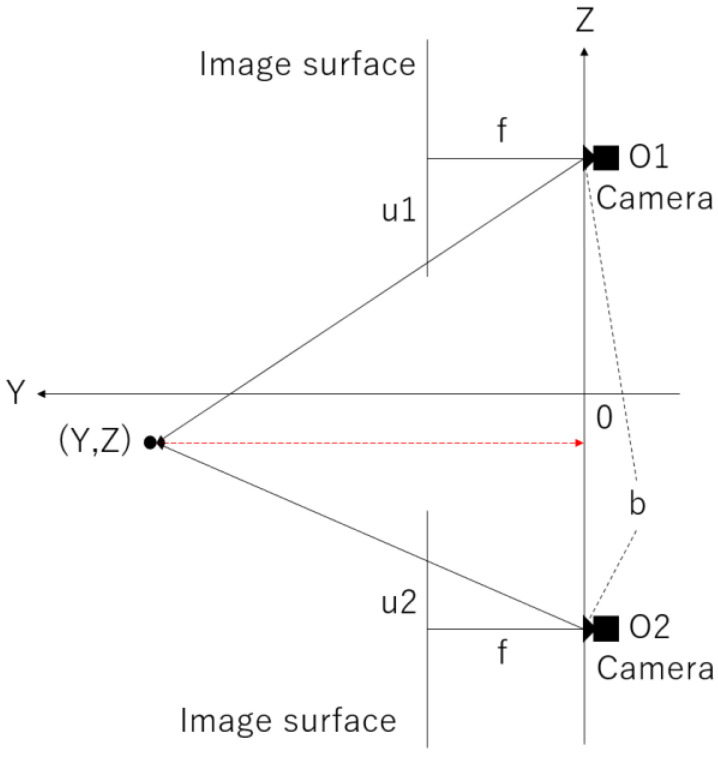
Conceptual diagram of a certain longitudinal cut plane.

The following is an explanation regarding the principle of stereo matching using vertical disparity. First, a diagram of Figure 2 considering a certain vertical cut plane is shown in Figure 12.

The focal distance f is between the upper and lower virtual panoramic cameras, the distance (baseline) b is between the virtual panoramic cameras, the object to be measured is at (Y,Z), the centers of the left and right panoramic images are O1 and O2, respectively, and the deviations from these positions are expressed as u1 and u2, respectively. From the similarity condition of the triangles, using the ratio

(4) b:u2−u1=Y:f

and solving for Y, we obtain

(5) $$Y=\frac{bf}{u2-u1}$$

This allows us to find the distance Y to the object.

## 3. Results

### 3.1. Purpose of the Experiment

Measurement experiments were conducted to verify the accuracy of this method. In addition, to verify the possibility of measuring in all directions, a depth image generation experiment of a panoramic image was conducted.

### 3.2. Measurement Experiment

#### 3.2.1. Experimental Method

In this experiment, the same experiment as in the previous study [10] was conducted for comparison with the previous study [10]. Target persons were photographed at 1.0 m intervals in the distance range of 1.0 m to 5.0 m from the omnidirectional stereo camera. Distance measurements were taken 20 times, and the average distance between the camera and the object was used as the experimental result. Relative error was used to evaluate accuracy; relative error is calculated by the following equation.

(6) $$RE=\frac{\left|AV-MV\right|}{AV}\times 100$$

*RE*: relative error

*AV*: actual value

*MV*: measured value

To confirm the measurement accuracy, we took measurements at five different points (1.00 m, 2.00 m, 3.00 m, 4.00 m, and 5.00 m) with 0.00 m directly underneath the device, and recorded the results.

The measurement range was determined based on the maximum speed of Toyota Motor Corporation’s fully automated vehicle, the e-Palette [3], as well as the stopping distance. The stopping distance was calculated as the sum of the empty run distance due to program processing time and the e-Palette’s braking distance.

The empty run distance is

(7) Empty run distance=reaction time [s]×car speed [m/s]

and the braking distance is determined by

(8) $$Breaking\;distance={\left(car\;speed\;\left[\mathrm{km}/h\right]\right)}^{2}\;÷\left(254\times coefficient\;of\;friction\right)$$

In this case, a coefficient of friction of 0.5 is assumed to account for bad weather conditions.

Since the maximum speed of the e-Palette is 5.28 (m/s) and the processing time of the current program is approximately 0.033 (s), the empty run distance is

(9) Empty run distance=5.28×0.033=0.17 [m]

Since the maximum speed of the e-Palette is 19 (km/h) and the coefficient of friction of the road in rain is 0.5, the braking distance is

(10) $$Breaking\;distance=19^{2}\;÷\;\left(254\times 0.5\right)=2.84\;\left[m\right]$$

Therefore, the total stopping distance including the empty run distance and the braking distance is approximately 3.01 (m). In addition, considering the size of the vehicle itself, the measurement accuracy of the distance up to 5 m is recorded in this case.

Details of the equipment used in the experiment are shown in Table 1.

The distance between cameras (baseline) b was set to 24.6 cm.

**Figure 13:**
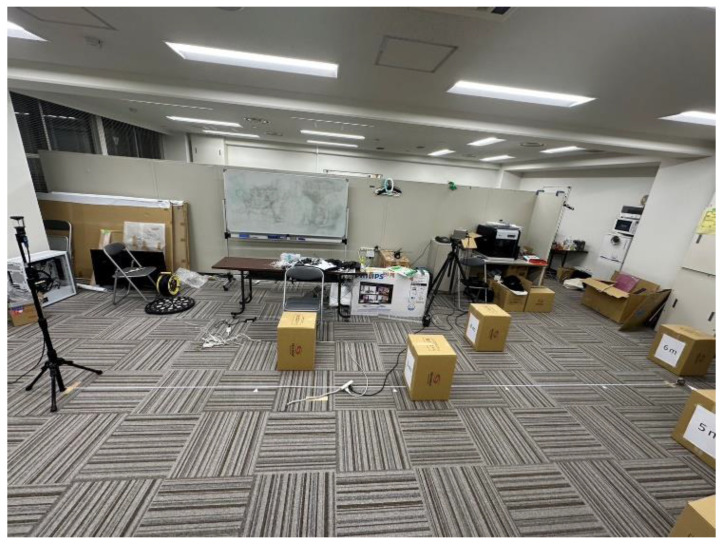
The experimental environment.

The experimental environment is shown in Figure 13.

**Figure 14:**
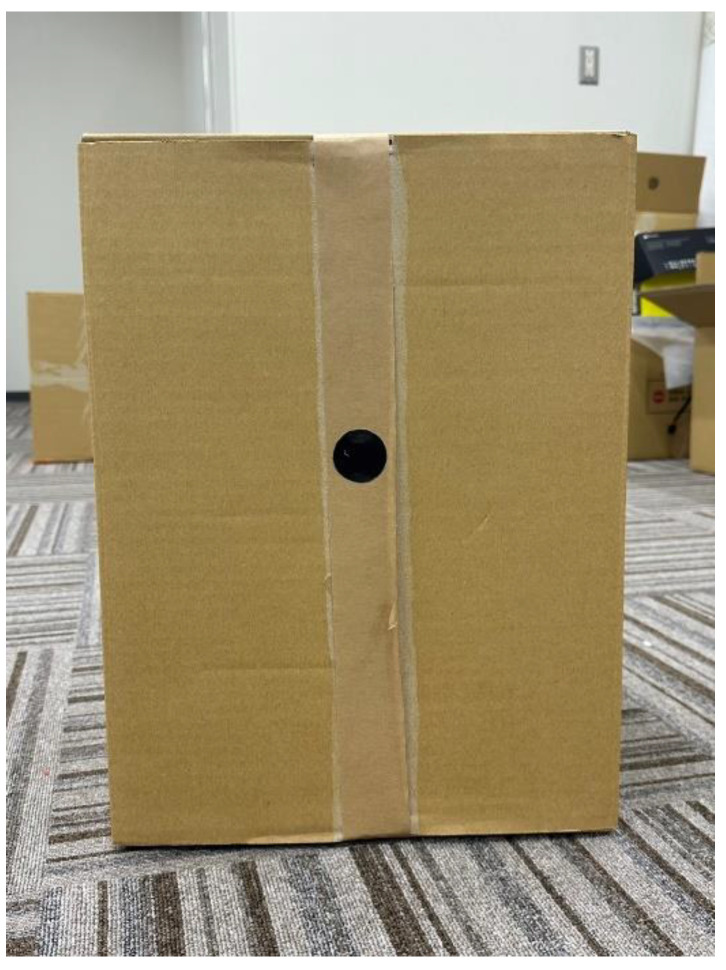
The object of measurement cardboard.

Measurements were taken at five points at distances of 1.00 m, 2.00 m, 3.00 m, 4.00 m, and 5.00 m from the camera. The object of measurement was the cardboard shown in the Figure 14. The correct distance was measured with a tape measure from directly under the camera.

#### 3.2.2. Experimental Results

**Figure 15:**
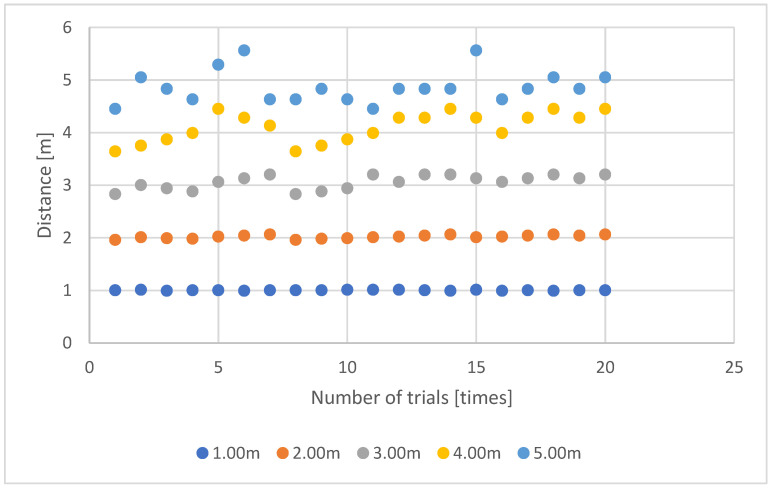
A graphical representation of the results.

The proposed method’s results are graphically depicted in Figure 15. Meanwhile, Table 2 summarizes the outcomes of the proposed method and the conventional method.

#### 3.2.3. Consideration

To validate the accuracy, the data were compared with data from a previous study. While the conventional method had a relative error of up to 40%, the new method succeeded in reducing the error to a maximum of 2.6%. In addition, the relative error for each distance was compared, and the accuracy was higher at all locations.

However, as shown in Figure 15, the stability of the measurements decreases as the distance from the camera increases. This is due to the characteristics of stereo measurement, where the accuracy is determined by the actual length of one pixel in the image, which is influenced by the lens, camera resolution, shooting distance, and baseline distance between cameras. In addition, since the stereo correspondence point pixel in the camera image plate has a size, the point to be measured can only be identified within a certain range in the actual space, which results in measurement errors. Typically, errors are larger in the depth direction from the camera. While lengthening the baseline can reduce the depth error, it can result in larger errors for shorter distances or even make measurements impossible. Therefore, stereo image measurement systems require appropriate baseline settings for the specific conditions in which they will be used. Thus, this issue can be resolved by adjusting the baseline according to the device being mounted.

These results demonstrate the effectiveness of this study in terms of accuracy.

### 3.3. Depth Image Generation Experiment

#### 3.3.1. Experimental Method

Since the accuracy was verified in Section 3.2, this experiment verifies whether omnidirectional measurement is possible. In order to confirm whether this method is capable of omnidirectional measurement, an experiment was conducted to generate a depth image of a panoramic image. The method used for depth image generation is called the SGM method [13].

#### 3.3.2. Experimental Results

**Figure 16:**
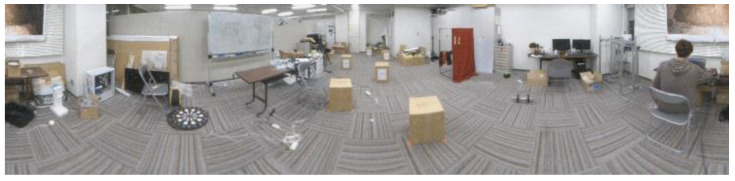
Original image.

**Figure 17:**
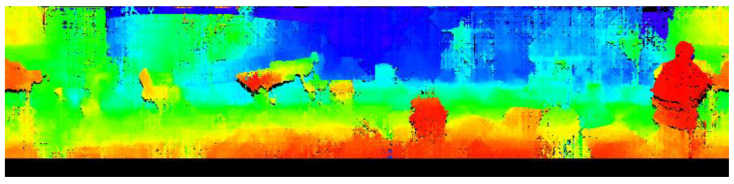
Depth image.

Figure 16 shows the original image and Figure 17 shows the depth image created.

For visual clarity, depth is replaced by hue. Undetected areas are output in black.

#### 3.3.3. Consideration

Through experiments, our proposed method was able to output an omnidirectional depth image that enabled confirmation of distances in all directions. It was confirmed that the system was able to detect obstacles such as humans, desks, chairs, boxes, and walls, indicating that it can be applied to autonomous driving. However, false detections were observed for featureless objects and areas, as well as for areas where the same features were repeatedly seen due to the reliance on block matching, which requires image features for detection. Despite this limitation, the primary goal of this project is obstacle detection and textured objects can be detected effectively. It can be used for obstacle detection with automatic guidance in the same way as the conventional stereo method. In the conventional method, it is impossible to measure in the complete omnidirectional direction as it includes the other camera in the image.

## 4. Discussion

We conducted two experiments to discuss the issues and potential solutions of the proposed system. From the measurement experiment of Measurement Experiment, we found that the system improved up to 37.5% in Relative error value compared to the conventional method. However, as mentioned in the Consideration, there was a problem with the lower measurement accuracy the further away the camera was from the object. To address this problem, we proposed changing the camera resolution, shooting distance, and baseline. By setting the baseline appropriately for the defined measurement range, we could expect stable measurements even in distant areas. However, a larger baseline would require a bigger device, which could result in a loss of mobility or design quality. Alternatively, increasing the camera resolution could improve depth direction accuracy but at a higher cost. Therefore, we concluded that the system should be flexible enough to change the baseline and camera parameters according to the machine’s size, maximum speed, and required measurement distance for autonomous driving. By doing so, we can ensure the system’s adaptability to different situations while maintaining accurate and stable measurements.

In Depth Image Generation Experiment, the proposed method successfully generated an omnidirectional depth image, allowing for distance checking in all directions. Additionally, the method detected various obstacles, including people, desks, chairs, boxes and walls, suggesting its potential use for autonomous driving. However, false detections were observed in featureless and repetitive areas, which is a common limitation of block matching that relies on image features. While the study did not consider this false detection as a significant issue since the main focus was obstacle recognition, further improvements are required if more precise detection is necessary. In this study, only feature points were used for detection, but we believe that the detection rate can be improved by using deep learning. Depth estimation methods using stereo vision with prior learning have been studied in recent years. Advances in image recognition technology, especially deep learning, have expanded research on depth estimation from images and videos. In depth estimation by stereo viewing using machine learning [14,15,16], stereo images captured by left and right cameras and correct depth maps (ground truth) acquired by LiDAR and millimeter wave radar are pretrained as teacher data. Stereo images captured under similar conditions are used as test data. By inputting the correct depth map (ground truth) obtained by LiDAR or millimeter wave radar as the teacher data, it is now possible to detect featureless areas and areas where the same features appear repeatedly, which has been difficult to achieve in the past. Therefore, in the future, the proposed method can be improved by using such a model to enhance the accuracy of depth images.

## 5. Conclusions

In this study, we proposed an omnidirectional measurement method with no blind spots by using a convex mirror, which in principle does not cause chromatic aberration, and by using vertical disparity by placing cameras above and below the image. Two experiments were conducted to demonstrate the effectiveness of this research, which achieved a wider measurement range and a smaller number of cameras while still allowing obstacle detection similar to the conventional stereo method.

Compared to the previous study [5], we achieved about twice the measurement range. Compared to the previous study [6], omnidirectional measurement is now possible with half the number of cameras. Moreover, compared to the previous studies [7,8,9,10], the new system enables complete omnidirectional measurement without any blind spots. Furthermore, the accuracy was improved by 37.4% compared to the previous study [10], which had the best accuracy among the studies that enabled measurement with two cameras. We consider this result to be a remarkable achievement in stereo measurement.

## Figures and Tables

**Table 1:** Details of the equipment used in the experiment.

Name of Equipment	Specification	
Camera	Name of maker	The Imaging Source (New Taipei City, Taiwan)
	Name of product	DFK 33 UX 183
	Name of sensor	Sony CMOS Exmor IMX 183 CQ
	Resolution	5472 × 3648
Lens	Name of maker	Shodensha Co., Ltd. (Osaka, Japan)
	Name of product	SM 1226–MP 20
	Focal length	12 mm
	Camera aperture range	F 2.6–F 16
Convex mirror	Name of maker	Vstone Co., Ltd. (Osaka, Japan)
	Name of product	VS–C 450 MR
	Mirror parameter a	29 mm
	Mirror parameter b	40 mm
	Mirror parameter c	49.4 mm
	Diameter of mirror	45 mm

**Table 2:** Experimental results of the proposed method and conventional method.

Distance between Omnidirectional Stereo Camera and Object (m)		Distance Measurement Results (m)		Mean Absolute Error (m)		Average Relative Error (%)	
Proposed Method	Conventional Method	Proposed Method	Conventional Method	Proposed Method	Conventional Method	Proposed Method	Conventional Method
1.00		1.00	1.20	0.00	0.20	0.0	20.0
2.00		2.02	1.60	0.02	0.40	1.0	20.0
3.00		3.06	2.90	0.06	0.10	2.0	3.3
4.00		4.10	5.60	0.10	1.60	2.5	40.0
5.00		4.87	6.30	0.13	1.30	2.6	26.0
